# Endogenous Mouse Dicer Is an Exclusively Cytoplasmic Protein

**DOI:** 10.1371/journal.pgen.1006095

**Published:** 2016-06-02

**Authors:** Christian Much, Tania Auchynnikava, Dinko Pavlinic, Andreas Buness, Juri Rappsilber, Vladimir Benes, Robin Allshire, Dónal O’Carroll

**Affiliations:** 1 Mouse Biology Unit, European Molecular Biology Laboratory (EMBL), Monterotondo, Italy; 2 MRC Centre for Regenerative Medicine, Institute for Stem Cell Research, School of Biological Sciences, University of Edinburgh, Edinburgh, Scotland, United Kingdom; 3 Wellcome Trust Centre for Cell Biology, School of Biological Sciences, University of Edinburgh, Edinburgh, Scotland, United Kingdom; 4 Genomics Core Facility, European Molecular Biology Laboratory (EMBL), Heidelberg, Germany; University of Cambridge, UNITED KINGDOM

## Abstract

Dicer is a large multi-domain protein responsible for the ultimate step of microRNA and short-interfering RNA biogenesis. In human and mouse cell lines, Dicer has been shown to be important in the nuclear clearance of dsRNA as well as the establishment of chromatin modifications. Here we set out to unambiguously define the cellular localization of Dicer in mice to understand if this is a conserved feature of mammalian Dicer *in vivo*. To this end, we utilized an endogenously epitope tagged Dicer knock-in mouse allele. From primary mouse cell lines and adult tissues, we determined with certainty by biochemical fractionation and confocal immunofluorescence microscopy that endogenous Dicer is exclusively cytoplasmic. We ruled out the possibility that a fraction of Dicer shuttles to and from the nucleus as well as that FGF or DNA damage signaling induce Dicer nuclear translocation. We also explored Dicer localization during the dynamic and developmental context of embryogenesis, where Dicer is ubiquitously expressed and strictly cytoplasmic in all three germ layers as well as extraembryonic tissues. Our data exclude a direct role for Dicer in the nuclear RNA processing in the mouse.

## Author Summary

Dicer has first been described as the enzyme dedicated to the generation of small RNA fragments from long double stranded RNA. This function of Dicer of playing a central role in the microRNA and short-interfering RNA biogenesis pathways was found to be taking place in the cytoplasm of the cell. However, recent studies reported additional functions of Dicer in the nucleus of human and mouse cell lines, where the protein was proposed to be involved in processing nuclear RNAs as well as in influencing the chromatin state. Consequently, the localization of Dicer within the cell has been highly debated ever since. In this study, we show by biochemical and microscopy techniques that Dicer is only localized in the cytoplasm of embryonic and adult tissues of the mouse. We also exclude the possibility that Dicer only transiently enters the cell nucleus. Our data indicate that nuclear RNA processing is not a conserved feature of mammalian Dicer and highlight the fact that findings from *in vitro* experiments in cell lines do not always predict the *in vivo* state within a living organism.

## Introduction

Dicer is a type III ribonuclease that catalyzes the ultimate biogenic step of several distinct classes of small regulatory non-coding RNAs across distinct phyla [1]. These small RNAs function in many aspects of genome and transcriptome regulation. MicroRNAs (miRNAs) are the primary Dicer products that are derived from genome encoded hairpin pre-miRNA structures that post-transcriptionally regulate gene expression [2]. In plants, they either pair with perfect or imperfect complementarity to post-transcriptionally regulate target gene expression [3,4]. In animals, miRNAs almost invariably pair imperfectly, culminating primarily in transcript destabilization through deadenylation [5–10]. The miRNA pathway is an essential post-transcriptional gene silencing system in both plants and animals.

Short-interfering RNAs (siRNAs) on the other hand are processed from dsRNA, where one strand is incorporated into an effector Argonaute protein [11,12]. In the fission yeast *S*. *pombe*, Dicer derived siRNAs direct the establishment and maintenance of centromeric heterochromatin [13]. In plants, siRNAs form an integral part of an adaptive immune system against viral pathogens and transposable elements [14]. This pathway is also present in mammals, where it is active in oocytes regulating gene/transposon expression [15,16], as well as in the soma, where Dicer processes dsRNA viral intermediates generating anti-viral siRNAs [17,18]. A defining feature of siRNAs is that they often pair with perfect complementarity to their targets and concomitantly induce their cleavage and destruction [11,19]. Dicer-catalyzed generation of siRNAs and miRNAs in mammals occurs in the cytoplasm [20].

Dicer is a large multi-domain protein. In addition to its two type III ribonuclease domains, most animal Dicer proteins contain a helicase and an RNA-binding domain [21]. Indeed, other non-canonical or non-miRNA/siRNA-generating functions for Dicer have recently revealed to be dependent and independent of its endonuclease activity [22–24]. Both human and *C*. *elegans* Dicer can function as a RNA binding protein (RBP) rather than a nuclease that can be recruited to hairpin-like structures in mRNAs as well as lncRNAs [23,24]. Interestingly, Dicer does not process these RNAs but can exert regulatory functions such as recruitment to P-bodies [23].

Mammalian Dicer was initially characterized as an exclusively cytoplasmic protein [25,26], but recent reports have challenged this localization [27]. Dicer was shown to interact with nuclear pore components and engage in nucleocytoplasmic shuttling [28]. An interaction of Dicer with ribosomal DNA repeats was demonstrated, however, a specific function could not be identified [29]. Furthermore, a large fraction of human Dicer was detected in the nucleus of HEK293 cells, where it was shown to cleave dsRNA, failure of which results in cell death due to the accumulation of dsRNA and consequent activation of the interferon response [30]. In both mouse and human cell lines, an additional nuclear role for Dicer was shown in the termination of transcription [31]. R-loops in the vicinity of a terminator induce antisense transcription and the formation of dsRNA that in turn recruits Dicer [31]. Dicer-mediated processing of this terminator-associated dsRNA results in the loading of Argonaute proteins with small RNAs and the subsequent recruitment of G9a and H3K9me2 at terminators, which reinforces RNA polymerase II (Pol II) pausing and transcriptional termination [31]. In addition, Dicer was identified as a regulator of alternative cleavage and polyadenylation of pre-mRNA in the nucleus of HEK293 cells [32]. Doyle *et al*. show that the double-stranded RNA binding domain (dsRBD) at the C-terminus of human Dicer has the potential to function as a nuclear localization signal (NLS). However, in full length Dicer, the NLS is masked and Dicer is shown to be cytoplasmic under steady-state conditions [33]. Different relative amounts of nuclear Dicer have been reported, with nuclear-cytoplasmic ratios ranging from approximately 1:4.3 [34] to 1:1.5 [35,36]. Given all the above studies were restricted to cell lines, we sought to explore the extent of nuclear Dicer function *in vivo* using the mouse as a model system. Surprisingly, we found that Dicer in the mouse is an exclusively cytoplasmic protein, indicating that nuclear RNA processing is not a conserved feature of mammalian Dicer *in vivo*.

## Results and Discussion

**Fig 1:**
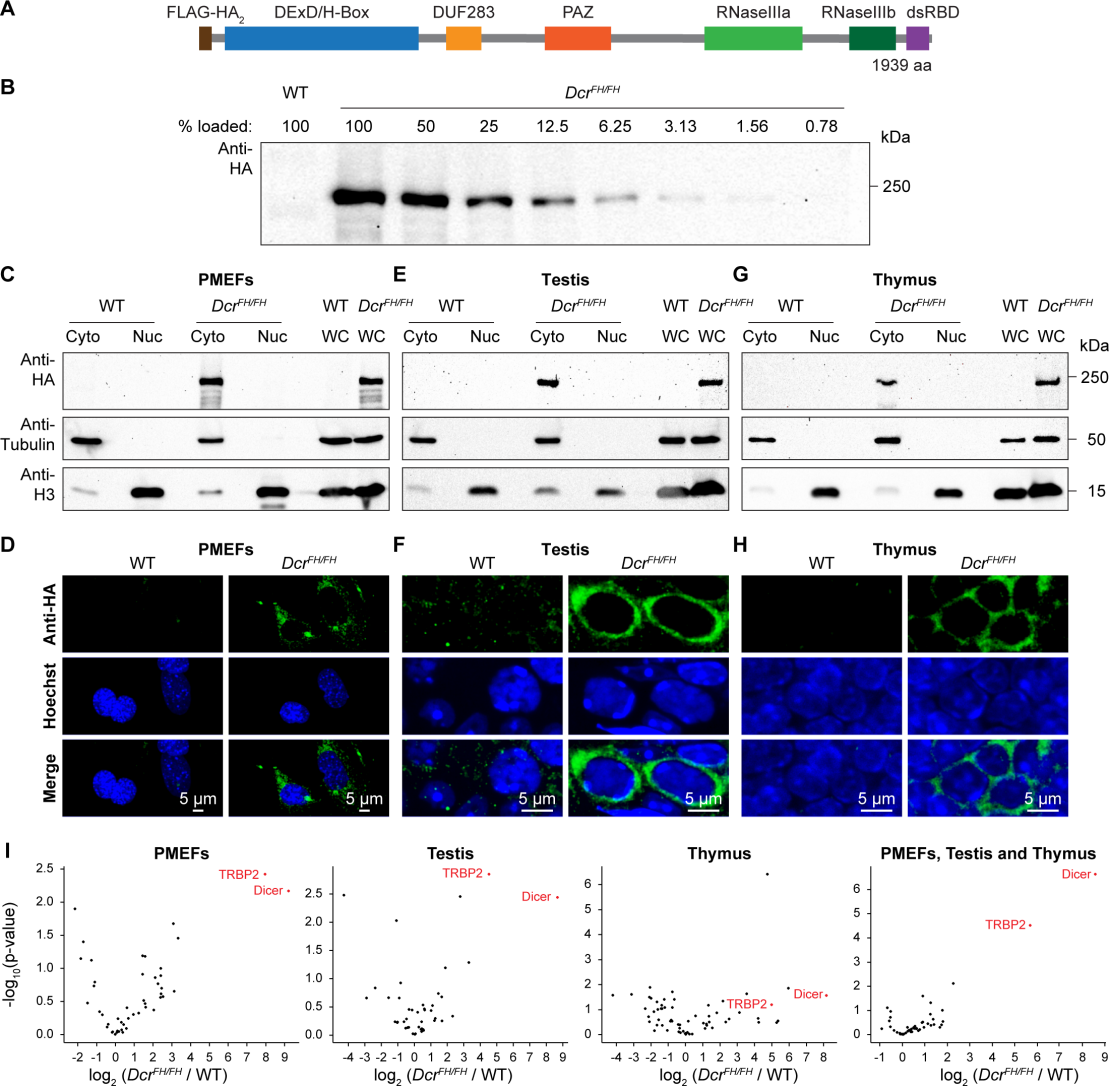
Dicer localizes exclusively to the cytoplasm of embryonic fibroblasts and adult tissues. (A) Schematic representation of the domain structure of N-terminal Flag-HA_2_ tagged murine Dicer. (B) Western blot using anti-HA antibody on serial dilution of whole cell extract from *Dcr*^*FH/FH*^ PMEFs. (C, E and G) Western blot analysis after subcellular fractionation using anti-HA antibody on cytoplasmic (Cy), nuclear (Nu) and whole cell (WC) extracts from wild type and *Dcr*^*FH/FH*^ PMEFs (C), adult testis (E) and thymus (G). Tubulin served as a cytoplasmic marker and histone H3 as a nuclear marker. (D, F and H) Confocal immunofluorescence micrographs showing wild type and *Dcr*^*FH/FH*^ PMEFs (D) as well as sections of adult testis (F) and thymus (H) stained with anti-HA antibody. Nuclei are stained with Hoechst. Scale bars represent 5 μm. (I) Immunoprecipitation using anti-HA beads and mass spectrometry analysis of the Dicer interactome in PMEFs, testis and thymus (each biological duplicates) and all of them pooled (six biological replicates).

In order to explore the nuclear function of Dicer *in vivo*, we first sought to determine the relative nuclear-cytoplasmic ratios in primary cell lines and in mouse tissues. The Dicer locus in mouse encodes two transcripts, one that is specific to oocytes and the other that is ubiquitously expressed. The oocyte transcript is driven by a MT-C retrotransposon insertion that is specific to rodents and generates a transcript that encodes a shorter protein lacking the N-terminal DExD helicase domain [37]. This truncated protein has fortuitously higher dsRNA processing activity resulting in the enhanced production of siRNAs for which oocyte development is uniquely dependent upon. To determine the localization of Dicer *in vivo*, we utilized a recently generated epitope-tagged allele of Dicer, where a Flag-HA-HA tag was placed after the start codon of the ubiquitously expressed transcript, resulting in N-terminally FH-tagged endogenous Dicer (FH-Dcr) (Fig 1A) [38]. In this study, we used tissues and primary cells derived from mice that were homozygous for the *Dcr*^*FH*^ allele in order to analyze all Dicer protein expressed in the cell that would not be possible in *Dcr*^*FH/+*^ cells/mice. A major advantage of the HA-epitope is that specific antibodies are available that work for multiple applications such as western blotting, immunoprecipitation (IP) and most importantly immunofluorescence (IF) on tissue sections. Firstly, we generated *Dcr*^*FH/FH*^ and control wild type primary mouse embryonic fibroblasts (PMEFs), prepared whole cell extracts and performed western blotting using an anti-HA antibody. To define the sensitivity of our Dicer western blotting, we performed a two-fold dilution series of the whole cell extract, from 100% to 0.78%. We could detect FH-Dcr in the lane with 1.56% of the loaded total whole cell extract, defining this as the detection limit of FH-Dcr for western blotting (Fig 1B). Next, we prepared nuclear and cytoplasmic extracts from *Dcr*^*FH/FH*^ and wild type PMEFs. For western blot analysis, protein amounts from the same number of cells were used for all fractions. The purity of the respective preparations was confirmed using antibodies recognizing the exclusively cytoplasmic tubulin and nuclear Histone H3 proteins (Fig 1C). Interestingly, endogenous FH-Dcr was only observed in the cytoplasmic fraction. Should the amount of potential nuclear Dicer be below our detection limit, it would amount for less than 1.56% of the whole cell extract. However, we cannot exclude the potential loss of nuclear soluble factors in the preparation of the nuclear fraction. Therefore, we applied confocal IF and confirmed the restriction of Dicer to the cytoplasm in the biochemical fractionation experiment (Fig 1D). Next we sought to determine the localization of Dicer in adult mouse tissues by the same methodologies. We selected mouse testis and thymus, as single cell suspensions can be readily made, which is essential for the sub-cellular fractionation protocol. Again, both by western blotting and confocal IF, Dicer was observed to be exclusively cytoplasmic (Fig 1E–1H). Importantly, quantification of confocal microscopy signals determined that the nuclear fluorescence intensity of FH-Dcr is lower than the autofluorescence of the wild type in PMEFs, testis and thymus (S1 Fig). Thus, mouse Dicer in these cell types displays an exclusively cytoplasmic localization.

While co-IP data demonstrate that human Dicer interacts with RNA Pol II [30] and engages in multiprotein complexes together with Ago2, TRBP and TNRC6A in the nucleus [35], fluorescence correlation spectroscopy suggests the existence of ectopically expressed EGFP-Dicer without any binding partner in the nucleus of human cell lines [34]. We therefore decided to analyze the Dicer interactome through IP coupled to mass spectrometry (MS) from PMEFs, testis and thymus whole cell extracts using anti-HA beads. This analysis revealed robust and highly significant interaction with Dicer’s interacting protein TRBP that recruits Ago2 [39] (Fig 1I). Interacting peptides for Ago2 were enriched in the Dicer IP but those for TNRC6A were not. However, we did not observe Dicer interaction with any components of RNA Pol II (S1–S4 Tables). Thus, the interaction of Dicer with RNA Pol II is not a conserved feature of mammalian Dicer.

**Fig 2:**
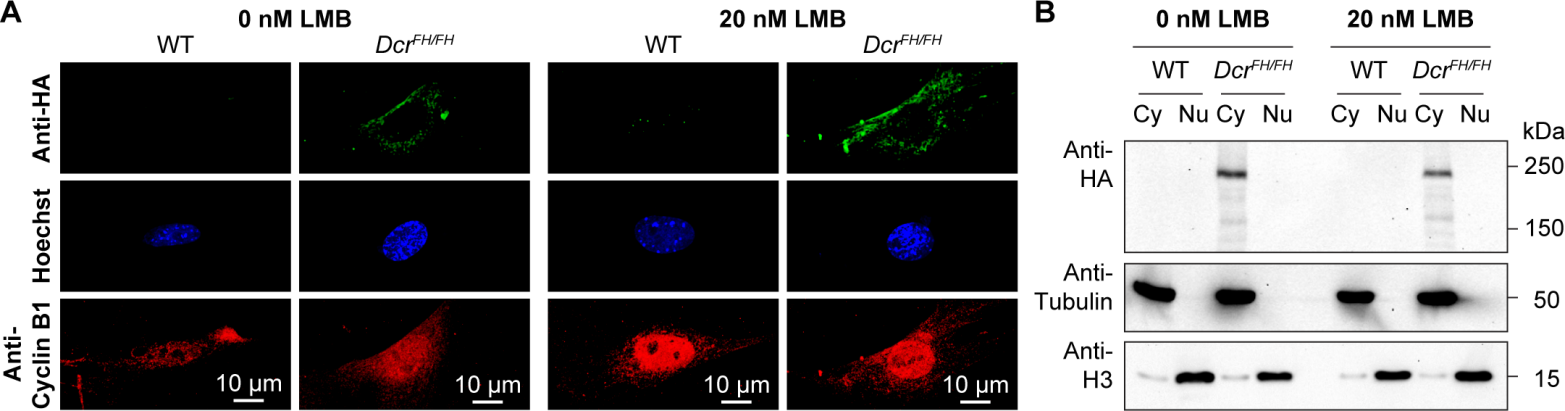
Dicer is not a nucleocytoplasmic shuttling protein. (A) Confocal micrographs showing immunofluorescence anti-HA staining of wild type and *Dcr*^*FH/FH*^ PMEFs without and with LMB-induced inhibition of nuclear export. Cells were treated with 20 nM LMB or solvent for 6 h. Cyclin B1 served as a positive control for the LMB treatment. Nuclei are stained with Hoechst. Scale bars represent 10 μm. (B) Western blot analysis after LMB treatment and subcellular fractionation. Anti-HA antibody was used on cytoplasmic (Cy) and nuclear (Nu) extracts, where tubulin served as a cytoplasmic marker and histone H3 as a nuclear marker.

The observation of Dicer residing solely in the cytoplasm of primary mouse cells and *ex vivo* isolated cells and tissues does not exclude the possibility that a tiny fraction of Dicer shuttles to and from the nucleus. To investigate this possibility, we treated PMEFs with leptomycin B (LMB) that is an inhibitor of nuclear export. Cyclin B1, a protein that is known for nuclear-cytoplasmic shuttling [40], became enriched in the nucleus of PMEFs after 6 h of LMB treatment (Fig 2A). However, Dicer remained exclusively cytoplasmic as determined by confocal IF (Fig 2A). Biochemical fractionation coupled with western blotting assays also revealed an exclusive cytoplasmic localization (Fig 2B). We therefore concluded that a small percentage of Dicer does not rapidly shuttle to and from the nucleus.

**Fig 3:**
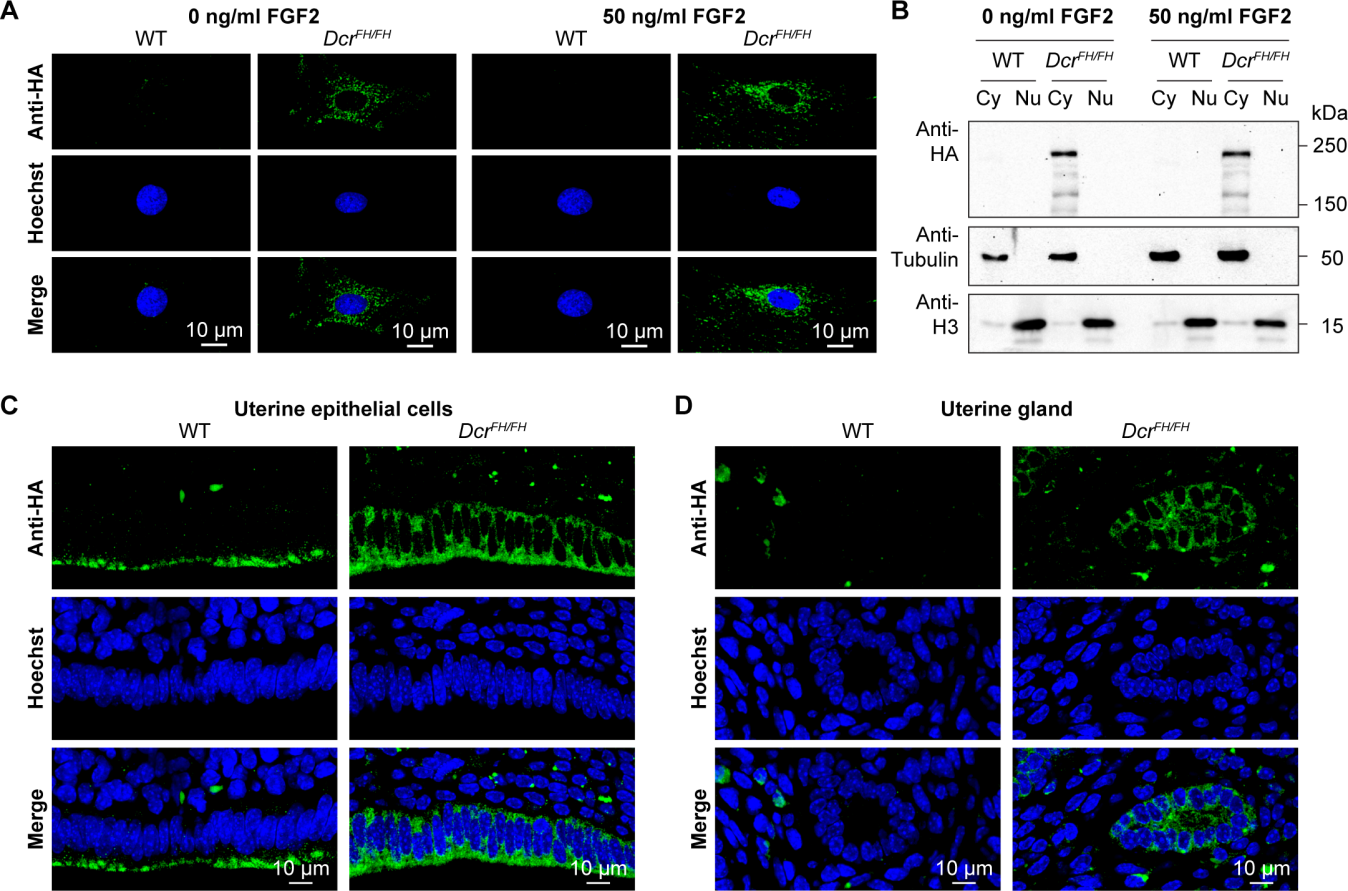
FGF signaling does not induce translocation of Dicer to the nucleus. (A) Confocal micrographs showing immunofluorescence anti-HA staining of wild type and *Dcr*^*FH/FH*^ serum starved PMEFs after stimulation with FGF2. Nuclei are stained with Hoechst. Scale bars represent 10 μm. (B) Western blot analysis after FGF2 stimulation and subcellular fractionation. Anti-HA antibody was used on cytoplasmic (Cy) and nuclear (Nu) extracts of treated and untreated cells. Tubulin is a cytoplasmic marker and histone H3 a nuclear marker. (C and D) Anti-HA immunofluorescence of wild type and *Dcr*^*FH/FH*^ uterus sections showing epithelial cells lining the lumen (C) and uterine glands (D).

In *C*. *elegans*, Dicer was identified to localize to the nucleus after ERK-dependent phosphorylation during the oocyte-to-embryo transition [41]. The phosphorylation sites of Dicer are conserved from worms to mammals, and nuclear phospho-Dicer was shown to be present in HEK293T cells upon stimulation with fibroblast growth factor (FGF) as well as in the mouse uterus [41]. To understand if FGF signaling can induce Dicer nuclear translocation, we stimulated PMEFs with FGF2 after serum starvation, but could not detect any nuclear Dicer, neither by confocal IF (Fig 3A) nor by western blotting after cellular fractionation (Fig 3B). Next we explored the localization of Dicer in the uterus by confocal IF. Consistent with our previous findings, Dicer was again restricted to the cytoplasm in the uterus (Fig 3C and 3D).

**Fig 4:**
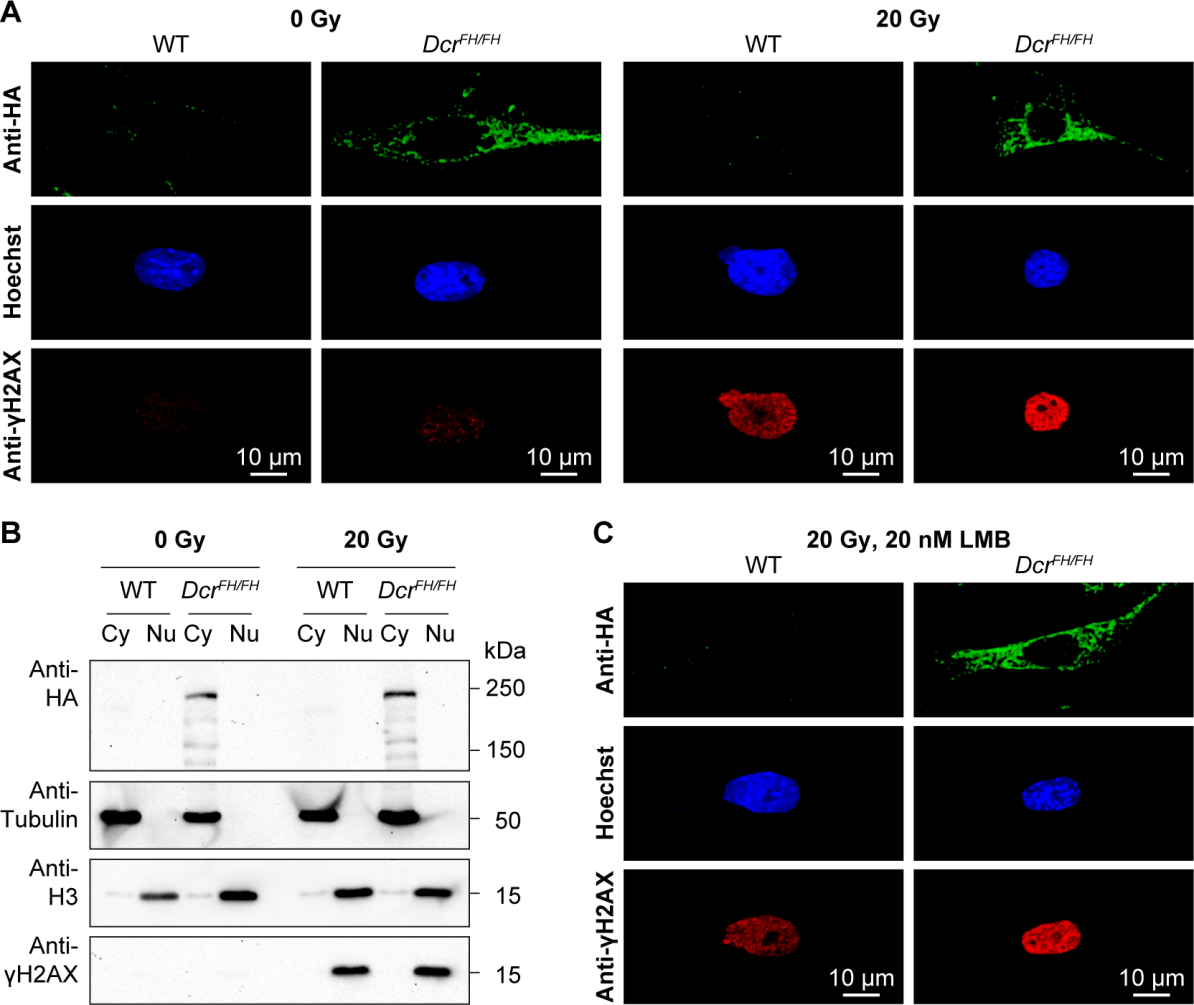
Dicer does not translocate to the nucleus after DNA damage. (A) Confocal imaging of anti-HA stained wild type and *Dcr*^*FH/FH*^ PMEFs after irradiation. Cells were irradiated with 20 Gy and then allowed to recover for 30 min. Activation of the DNA damage response was verified with anti-γH2AX staining. Nuclei are stained with Hoechst. Scale bars represent 10 μm. (B) Western blot analysis after irradiation and subcellular fractionation. Anti-HA antibody was used on cytoplasmic (Cy) and nuclear (Nu) extracts of irradiated and non-irradiated cells. Tubulin served as a cytoplasmic marker, histone H3 as a nuclear marker and γH2AX as a marker of DNA damage. (C) Confocal microscopy on anti-HA stained wild type and *Dcr*^*FH/FH*^ PMEFs after irradiation and LMB treatment.

Dicer and a class of Dicer-dependent small RNAs termed DNA damage response RNAs (DDRNAs) also function in DNA repair, however, the subcellular localization of DDRNA processing is not known [42]. Although from our observations thus far DDRNAs are likely made in the cytoplasm, one cannot exclude the possibility that DNA damage signaling results in the translocation of Dicer to the nucleus. We therefore treated MEFs with 20 Gy of irradiation and analyzed Dicer localization 30 min after. The broad accumulation of γH2AX in the nucleus of irradiated cells confirmed extensive DNA damage (Fig 4A and 4B). Both western blotting of Dicer in cytoplasmic and nuclear fractions as well as confocal IF revealed that DNA damage signaling does not alter Dicer’s cytoplasmic localization (Fig 4A and 4B). A caveat of this experiment is that the translocation to the nucleus may be very transitory and we may have missed it with our single 30-min post-irradiation time point. We therefore treated the irradiated cells with 20 nM LMB to block nuclear export and trap any Dicer that may have translocated into the nucleus. Blocking nuclear export did not result in the accumulation of Dicer in the nucleus upon DNA damage (Fig 4C). In summary, DNA damage signaling does not recruit Dicer to the nucleus and therefore DDRNA biogenesis must be a cytoplasmic event.

**Fig 5:**
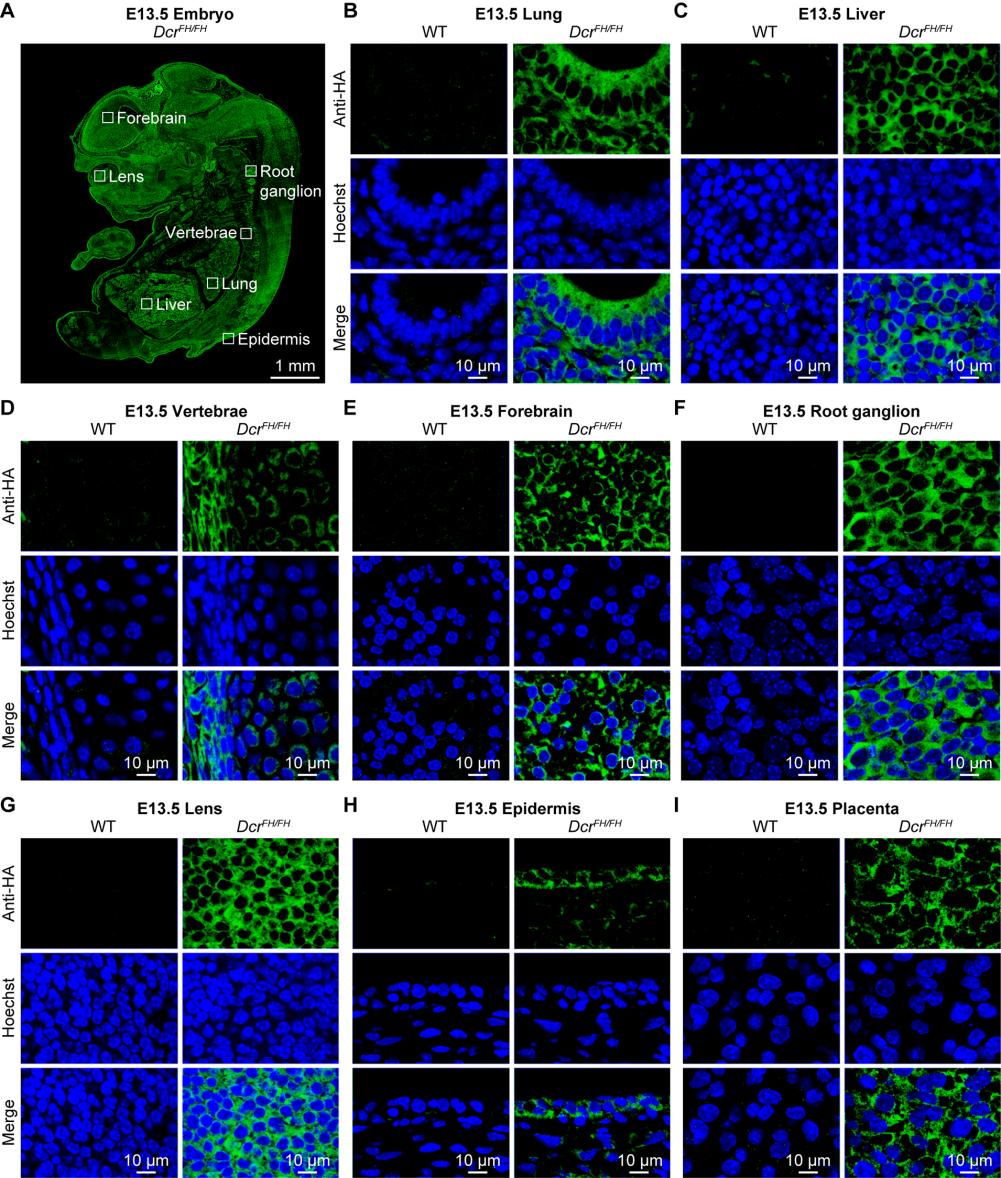
Dicer is solely cytoplasmic during embryogenesis. (A) Anti-HA immunofluorescence of E13.5 *Dcr*^*FH/FH*^ embryo section. Scale bar is 1 mm. (B-I) Confocal microscopy of immunofluorescence staining of wild type and *Dcr*^*FH/FH*^ E13.5 tissue sections with anti-HA antibody. Sections of lung (B) and liver (C) are shown as representatives of endoderm, vertebrae (D) represent mesoderm, forebrain (E), root ganglion (F), lens (G) and epidermis (H) represent ectoderm, placenta (I) is depicted as an example for an extraembryonic tissue. Nuclei are stained with Hoechst. Scale bar is 10 μm.

Our experiments would suggest that at least in PMEFs as well as in adult thymus, testis and uterus, Dicer is exclusively cytoplasmic. This is a small representation of adult mouse tissues and embryonic cells, we thus sought to extend our analysis to other tissues and incorporate the demanding and dynamic nature of embryonic development. Therefore, we performed an extensive analysis of Dicer localization across the embryonic day 13.5 (E13.5) mouse embryo. We chose this stage of mid-gestation development, as all organs/tissues are specified and the entire embryo can also be analyzed by IF on single sagittal sections. Staining of FH-Dcr E13.5 sagittal sections revealed as expected Dicer to be a ubiquitously expressed protein, although the levels of expression varied slightly between tissues (Fig 5A). We analyzed derivatives of all three germ layers. Within the endoderm derived fetal lung and liver (Fig 5B and 5C), Dicer was cytoplasmic. The same applied to mesodermal vertebrae (Fig 5D) as well as the ectoderm-derived forebrain, root ganglion, lens, and epidermis (Fig 5E–5H). Likewise, extraembryonic tissues such as the placenta featured a solely cytoplasmic localization of Dicer (Fig 5I). Every tissue within the E13.5 embryo that was analyzed by confocal microscopy gave the same exclusively cytoplasmic Dicer staining. Although *dicer* has one described somatic transcript in the RefSeq databases, an alternative transcription start site (TSS) that would result in a truncated Dicer protein not possessing the FH-tag could exist. To exclude this unlikely eventuality, we subjected total RNA from wild type and *Dcr*^*FH/FH*^ E13.5 mouse embryos to global 5’ rapid amplification of cDNA ends (5’RACE) followed by high throughput sequencing. Our results demonstrate that in E13.5 mouse embryos, the d*icer* transcript originates from a single TSS (S2 Fig). In summary, we conclude that Dicer is an exclusively cytoplasmic protein during mid gestation mouse embryonic development.

Here we show that Dicer *in vivo* in the mouse is an exclusively cytoplasmic protein, excluding a conserved role for Dicer in nuclear RNA processing. This raises the possibility that Dicer-mediated nuclear clearance of dsRNA and thus restriction of deleterious interferon responses may not be a general feature of mammalian RNA biology. In contrast, the reported function of Dicer in metabolizing terminator-associated R-loop-induced dsRNA and the concomitant recruitment of repressive chromatin marks was shown both in human and mouse cell lines. Should Dicer-induced chromatin changes be central *in vivo* for the termination of transcription, one would expect to see a fraction or even sizable portion of Dicer in the nucleus. This discrepancy may arise from the fact that cell line observations may not always reflect the mechanism *in vivo* in an animal model. Given that Dicer was not observed in the nucleus with any of our methods, a physiological relevant role of murine nuclear Dicer at amounts below the detection limit seems unlikely *in vivo*. In summary, we show that mouse Dicer *in vivo* both in PMEFs and adult tissues as well as the developing embryo is a cytoplasmic protein restricting its physiological function to cytoplasmic RNA metabolism, binding and regulation.

## Materials and Methods

### Ethics statement

Mice were bred and maintained at the EMBL Mouse Biology Unit in Monterotondo in accordance with current Italian legislation (Art. 9, 27. Jan 1992, nu116) under license from the Italian health ministry.

### Mice

The *Dcr*^*FH*^ allele has been described previously [38].

### PMEF isolation and culture

PMEFs were derived from E13.5 embryos of *Dcr*^*FH/+*^ intercrosses according to standard protocols. Cells were cultured in DMEM supplemented with 12.5% fetal calf serum, 2 mM L-glutamine, 1X non-essential amino acids, 100 units/ml penicillin/streptomycin and 100 μM β-mercaptoethanol (all Gibco) at 37°C and 7.5% CO_2_.

### Antibodies

Anti-HA (Covance, MMS-101P) was used at 1:1000 for WB and 1:100 for IF, anti-HA (Cell Signaling, 3724) at 1:500 for IF, anti-α-tubulin (Sigma, T9026) and anti-histone H3 (Abcam, an1791) at 1:1000 for WB, anti-cyclin B1 (Santa Cruz, sc-752) at 1:100 for IF and anti-γH2AX (Abcam, ab26350 and Bethyl, IHC-00059) at 1:1000 for WB and IF.

### Subcellular fractionation and western blot analysis

Cytosolic extracts were prepared by incubating cells in CSK buffer (0.5% Triton X-100, 100 mM NaCl, 3 mM MgCl_2_, 300 mM sucrose, 1 mM EGTA, 1 mM Pipes pH 6.8, protease inhibitors) for 10 min on ice. Supernatants were recovered after centrifugation at 500 g for 4 min. Nuclear extracts were prepared by pelleting nuclei by centrifugation of cells with increasing speed of 20 g in 30 s intervals from 20 g to 100 g trough a cushion of nuclear isolation buffer (20% Ficoll-Paque, 80 mM Tris pH 7.4, 8 mM MgCl_2_, 8 mM CaCl_2_, 1.6% Triton X-100, 0.1% DMSO). The nuclei pellet was washed in PBS, resuspended in Laemmli sample buffer and boiled for 10 min at 95°C. Whole cell extracts were generated by resuspending the cell pellet in Laemmli sample buffer and boiling for 10 min at 95°C. Proteins from the same number of cells for every fraction were separated on 5% and 12% SDS-PAGE gels, respectively, and transferred overnight onto a nitrocellulose membrane (GE Healthcare) by wet transfer. The membrane was blocked with 3% milk/0.1% Tween-20 in PBS, incubated in primary antibody overnight, washed, and incubated with appropriate secondary antibody linked to horseradish peroxidase (Sigma). Proteins were detected using the ECL Western Blotting Detection Reagent (Amersham).

### Leptomycin B treatment

Nuclear export was inhibited with LMB (LC Laboratories). PMEFs were treated with 20 nM LMB (dissolved in ethanol) for 6 h. Control cells were incubated with same amounts of ethanol instead.

### FGF treatment

PMEFs were grown to 50–60% confluency, starved in medium without FCS for 16 h and then treated with 50 ng/ml FGF2 (Peprotech) for 30 min.

### Irradiation

PMEFs were irradiated for 10 min with a total dose of 20 Gy using a RS 2000 biological irradiator (Rad Source Technologies) and then incubated at 37°C for another 30 min.

### Immunofluorescence staining

Cells were seeded onto glass coverslips, fixed in 4% formaldehyde for 10 min and permeabilized in 0.5% NP-40 for 10 min at room temperature. Blocking was performed in PBG buffer (0.2% cold water fish gelatin, 0.5% BSA) for 1 h at room temperature, followed by incubation in primary antibody in PBG overnight at 4°C.

Tissues were collected and fixed in 4% formaldehyde overnight and embedded in paraffin. Sections of 7 μm thickness were subjected to antigen retrieval using steam vapor for 20 min in antigen unmasking solution (Vector Lab) and then permeabilized in 0.1% Triton X-100 for 10 min at room temperature. Sections were blocked in 10% donkey serum, 2% BSA and 0.1 M glycine (all Sigma) for 1 h at room temperature. Incubation in primary antibody was performed in blocking buffer overnight at 4°C. For both cells and tissue sections, appropriate Alexa secondary antibodies (Invitrogen) were used at 1:1000. Hoechst 33342 at 5 mg/ml (Sigma) was used to stain nuclei. Leica TCS SP5 confocal microscope was used to acquire z-stacks of four optical sections each of 0.5 μm thickness. Images of control and experimental samples were equally processed with ImageJ and Photoshop.

### Immunoprecipitation and mass spectrometry

Tissues were homogenized by douncing and incubated in IP extraction buffer (50 mM Tris pH 8, 150 mM KCl, 5 mM MgCl_2_, 0.2% Triton X-100, protease inhibitors) for 10 min on ice. After centrifugation at 14,000 g for 10 min, supernatants were recovered and incubated with Pierce anti-HA magnetic beads (Thermo Scientific, 88836) for 30 min at 4°C. Beads were washed four times with IP extraction buffer and two times with 50 mM Tris pH 8, 0.2% Triton X-100 and proteins were eluted in 50 mM Tris pH 8, 0.5% SDS for 15 min at 50°C. Biological replicas were trypsin digested as described [43], desalted using STAGE tips [44], resuspended in 0.1% TFA (v/v) and analyzed by nanoLC MS/MS. For the analysis, peptides were separated on an EasySpray (Thermo Scientific) 50 cm column coupled to Orbitrap Fusion Lumos (Thermo Scientific) mass spectrometer. Raw data were processed using MaxQuant version 1.5.2.8. Label-free quantitation was performed using MaxQuant LFQ algorithm [45]. Peptides were searched against mouse Uniprot database with commonly observed contaminants (trypsin, keratins). For visualization, LFQ intensities (output of MaxQuant search) were imported into Perseus version 1.5.1.6 and processed as described [46].

### Global 5’ RACE

Experimental protocol and bioinformatics analysis for the global 5’ RACE were performed as described [47].

### Accession numbers

The global 5’ RACE sequencing data are submitted to the European Nucleotide Archive (ENA) under the accession number PRJEB13567. The original Leica SP5 files are provided under the DOI 10.5281/zenodo.49840.

## Supporting Information

S1 Fig Fluorescence intensity quantification. (A) Bar charts showing quantification of fluorescence intensities in the nucleus and cytoplasm of wild type and *Dcr*^*FH/FH*^ PMEFs, testis and thymus as determined by ImageJ. Background fluorescence was measured in an empty area of the microscopy slide. (B) Example confocal images depicting the area that was measured to calculate fluorescence intensities.
(TIF)

S2 Fig Dicer posseses only one transcription start site. Global 5’RACE was performed on wild type and *Dcr*^*FH/FH*^ E13.5 mouse embryo total RNA and reads from high throughput sequencing were mapped to the d*icer* locus. Dicer’s location on chromosome 12 is highlighted in red. The transcript of *dice*r is depicted in blue, the first coding exon, 5’ of which the FH-tag is placed, is indicated in green.
(TIF)

S1 Table Mass spectrometry data for PMEFs. (XLSX)

S2 Table Mass spectrometry data for testis. (XLSX)

S3 Table Mass spectrometry data for thymus. (XLSX)

S4 Table Mass spectrometry data for PMEFs, testis and thymus. (XLSX)
