# Supplementary figures and images for: Endogenous Mouse Dicer Is an Exclusively Cytoplasmic Protein
Source: PLoS Genet. 2016 Jun 2;12(6):e1006095. doi: 10.1371/journal.pgen.1006095 (PMC4890738; doi:10.1371/journal.pgen.1006095)

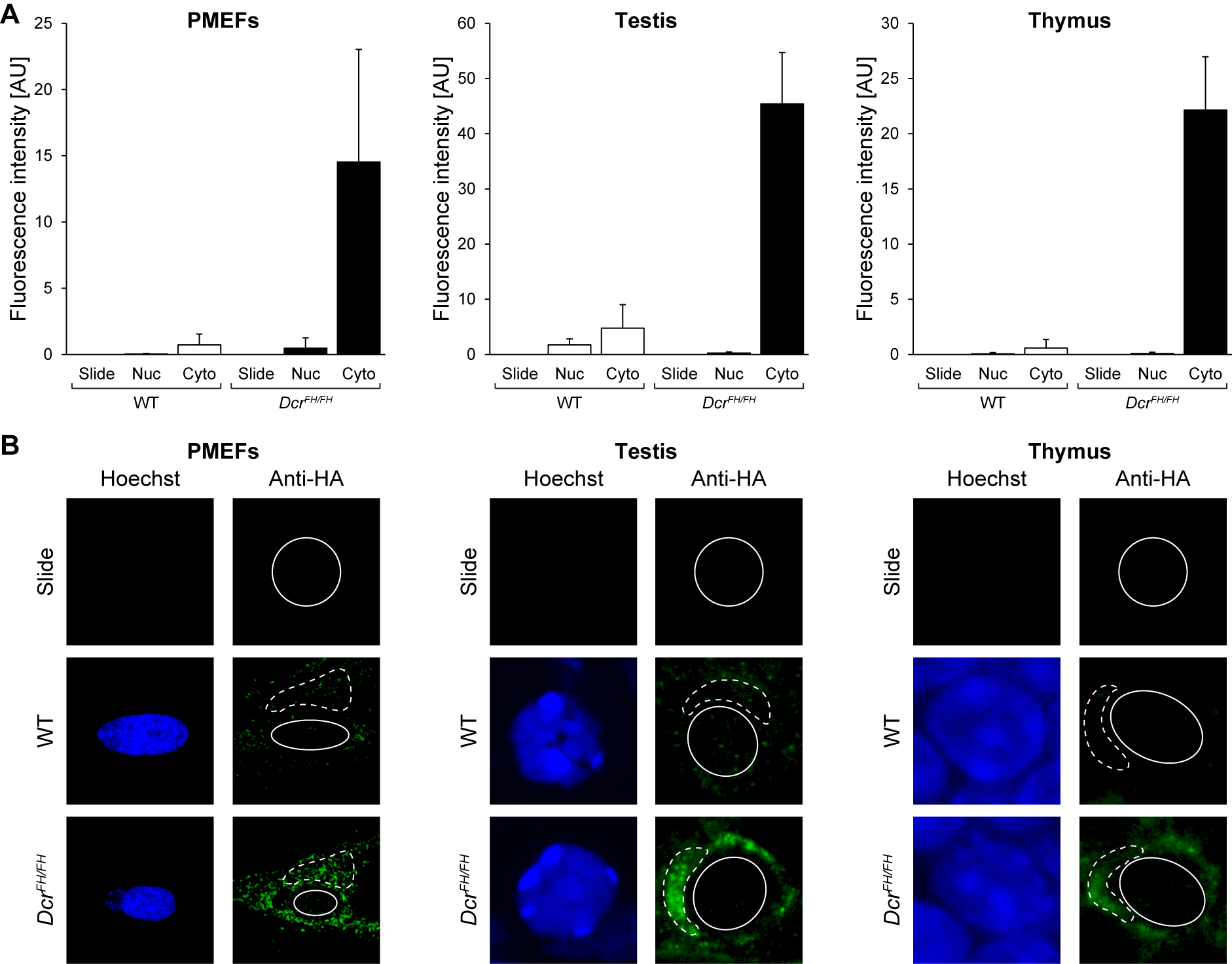

Supplement: S1 Fig — (A) Bar charts showing quantification of fluorescence intensities in the nucleus and cytoplasm of wild type and DcrFH/FH PMEFs, testis and thymus as determined by ImageJ. Background fluorescence was measured in an empty area of the microscopy slide. (B) Example confocal images depicting the area that was measured to calculate fluorescence intensities. (TIF) [file pgen.1006095.s001.tif]

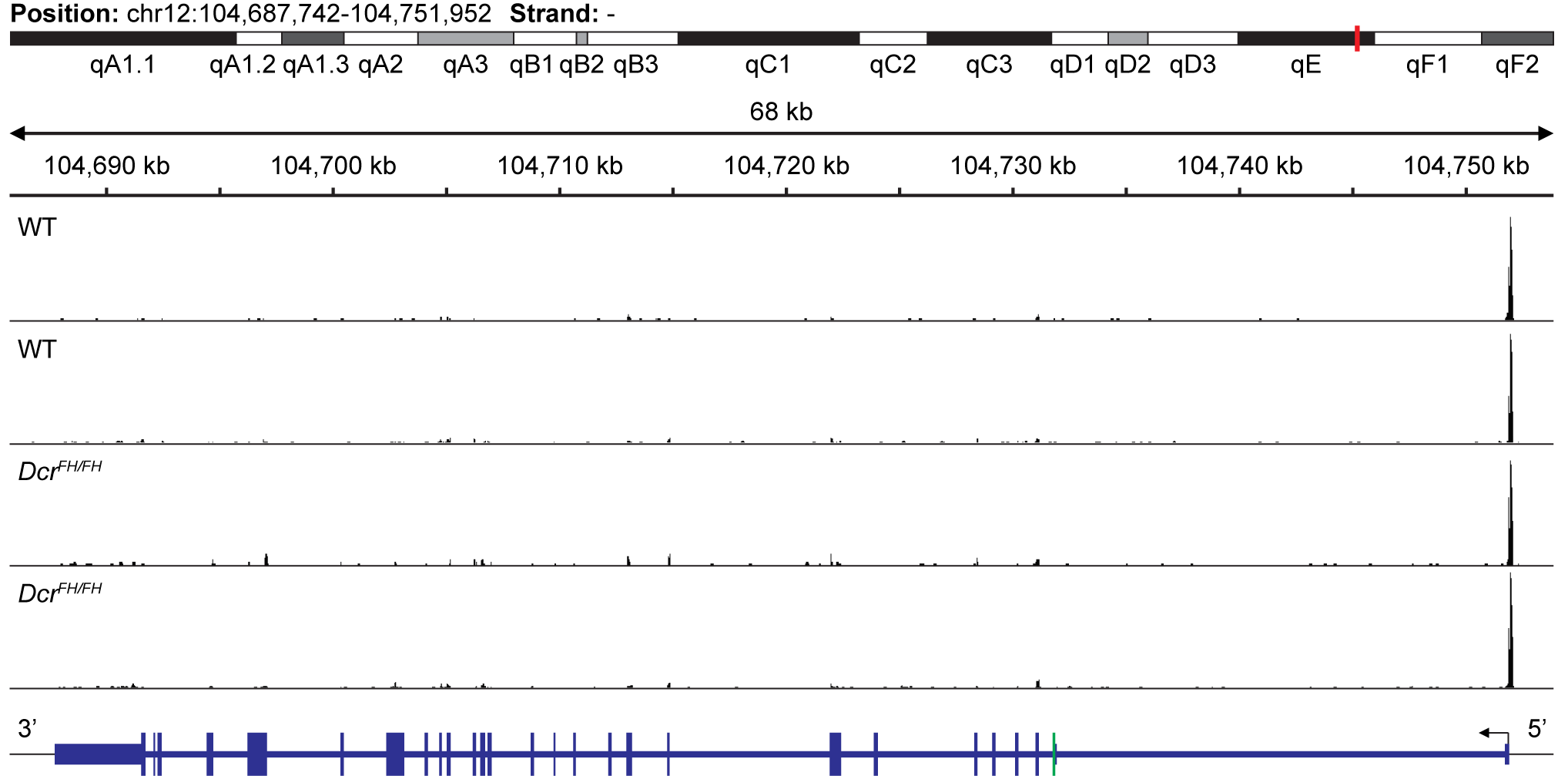

Supplement: S2 Fig — Global 5’RACE was performed on wild type and DcrFH/FH E13.5 mouse embryo total RNA and reads from high throughput sequencing were mapped to the dicer locus. Dicer’s location on chromosome 12 is highlighted in red. The transcript of dicer is depicted in blue, the first coding exon, 5’ of which the FH-tag is placed, is indicated in green. (TIF) [file pgen.1006095.s002.tif]
